# Relationships between depression, self-efficacy, and professional values among Chinese oncology nurses: a multicenter cross-sectional study

**DOI:** 10.1186/s12912-023-01287-9

**Published:** 2023-04-27

**Authors:** Jianfei Xie, Xiaofei Luo, Yi Zhou, Chun Zhang, Lijun Li, Panpan Xiao, Yinglong Duan, Qinqin Cheng, Xiangyu Liu, Andy SK Cheng

**Affiliations:** 1 Nursing Department, The Third Xiangya Hospital, Central South University, Changsha, Hunan China; 2 Xiangya Nursing School, Central South University, Changsha, Hunan China; 3 Hunan Cancer Hospital, Changsha, Hunan China; 4 Department of Rehabilitation Sciences, The Hong Kong Polytechnic University, Hong Kong, China

**Keywords:** Nurses, Oncology, Depression, Self-efficacy, Professional values

## Abstract

**Objectives:**

Many factors are related to oncology nurses’ professional values. However, the evidence on the relevance of professional values among oncology nurses in China remains sparse. This study aims to investigate the relationship between depression, self-efficacy, and professional values among Chinese oncology nurses and analyze the mediating effect of self-efficacy on this association.

**Methods:**

It was a multicenter cross-sectional study designed with the STROBE guidelines. An anonymous online questionnaire recruited 2530 oncology nurses from 55 hospitals in six provinces of China between March and June 2021. Measures included self-designed sociodemographic and fully validated instruments. Pearson correlation analysis was employed to explore the associations between depression, self-efficacy, and professional values. Bootstrapping analysis by the PROCESS macro was used to examine the mediating effect of self-efficacy.

**Results:**

The total scores of depression, self-efficacy, and professional values of Chinese oncology nurses were 52.75 ± 12.62, 28.39 ± 6.33, and 101.55 ± 20.43, respectively. About 55.2% of Chinese oncology nurses were depressed. Chinese oncology nurses’ professional values were generally intermediate. Their professional values were negatively related to depression and positively correlated with self-efficacy, while depression was negatively related to self-efficacy. Moreover, self-efficacy partially mediated the relationship between depression and professional values, accounting for 24.8% of the total effect.

**Conclusions:**

Depression negatively predicts self-efficacy and professional values, and self-efficacy positively predicts professional values. Meanwhile, depression in Chinese oncology nurses has an indirect effect on their professional values through self-efficacy. Nursing managers and oncology nurses themselves should develop strategies aimed at relieving depression and improving self-efficacy to strengthen their positive professional values.

**Supplementary Information:**

The online version contains supplementary material available at 10.1186/s12912-023-01287-9.

## Introduction

Nursing is known to be a valuable talent in healthcare and is vital to the health of patients. However, a nursing shortage is occurring worldwide [1]. Currently, as the incidence of all types of tumors increases, the role of oncology nurses is becoming more critical, and the professional challenges they face are becoming more severe, seriously affecting their perception of professional values [2, 3]. Nurses’ sense of professional values is closely related to their professional existence [4]. Improving the professional emotional experience of oncology nurses, promoting their professional adaptation potential, stabilizing the nursing talent team, and meeting the social demand for nursing services are essential issues concerning the construction and development of oncology specialist nurses.

Professional values are the subject’s self-judgment of their career values, the estimation of possible achievements, and the satisfaction degree of social return, which are the core factors affecting the subject’s occupational living state [5]. Research shows that professional values can stimulate an individual’s identity and sense of belonging to a career and improve their work enthusiasm, work efficiency, and job satisfaction [6, 7]. As an internal motivation factor, nurses’ professional values affect their physical and mental health and professional development [8, 9]. Nurses with a high sense of professional values will inject their own positive emotions into their careers, constantly improve themselves at work, and have a low tendency to resign [9, 10]. Given the specific characteristics of oncology nursing, it has been shown that oncology nurses suffer from more work-related problems and high levels of burnout than other types of nurses [11, 12]. Therefore, improving oncology nurses’ sense of professional values is vital for nursing managers to consider. Individual factors and internal and external working environments can affect the professional values of nurses [13]. In addition, research points out that nurses’ emotional states have affected their professional values [14].

Depression, as an adverse emotional state, can seriously disturb a person’s life and work [15, 16]. Due to the concentration of oncology patients and the high frequency of chemotherapy drug exposure, oncology nurses are constantly exposed to the refractory and negative emotions of patients and their families [17, 18]. This particular working environment and service focus make it inevitable for oncology nurses exposed to heightened professional risks for extended periods, making them prone to depression in their work [19, 20]. More importantly, an imbalance in nurses’ mental health will decrease their core workability, affect their professional values and create burnout [2, 21, 22]. The mental health and professional values of oncology nurses demand attention. It has been found that internal factors such as self-efficacy play a vital role in developing depression [23].

Self-efficacy is a concept introduced by the American psychologist Bandura [24] in 1977. It refers to an individual’s expectation of their ability to perform a behavior, representing the perception and evaluation of one’s ability to behave. Nurses with a high level of self-efficacy can exercise adequate self-control, regulate their performance and promote professional behavior in nursing practice [25].

Previous studies have found a significant negative correlation between self-efficacy and depression [26, 27]. This indicates that nurses’ psychological status may affect their self-efficacy. Meanwhile, self-efficacy has also been related to professional values [28]. Jun and Lee [29] pointed out that self-efficacy was the main factor influencing nurse students’ professional values. A correlational descriptive study in Italian also stated that nurses’ self-efficacy was significantly associated with professional values [30]. This suggests that the role of self-efficacy in the mental health and professional values of oncology nurses should be emphasized.

However, despite the valuable contribution of previous research, the exact relationships between depression, self-efficacy, and professional values remain unclear. In addition, most research on professional values in China has been conducted on nursing students or nurses as a whole [31]. The professional values of oncology nurses and their associated factors have not been explored in depth. Only by identifying these pathways of influence and the current situation can we better understand the professional values of oncology nurses and target interventions. Therefore, we hypothesized that self-efficacy would mediate the relationship between depression and professional values in oncology nurses.

The theoretical models that guided this study were cognitive-behavioral and career self-efficacy theories [32]. In cognitive-behavioral theory, cognition plays a mediating and coordinating role among emotion and behavior. Career self-efficacy theory is a specific application of self-efficacy theory in the career field, which believes that self-efficacy influences the psychological processes that motivate individuals’ careers, including career interests, career behavior, and other aspects [33]. Self-efficacy as a cognitive process was negatively associated with depression and affected feelings of professional values [27, 30]. Furthermore, depression as an antecedent was related to professional values as a consequence, which is both intrinsic to motivating behavior and can be directly expressed in the behavior itself [34].

As a result, this study intends to (1) inform Chinese oncology nurses’ depression, self-efficacy, and professional values and analyze the relationship between them, and (2) determine the mediating effect of self-efficacy on depression and professional values to provide a practical reference basis for effective oncology nursing management and practice.

## Methods

### Study design

This study used a multicenter cross-sectional study. The Strengthening the Reporting of Observational Studies in Epidemiology (STROBE) checklist was chosen to conduct and report [35].

### Participants

An online survey was conducted among oncology nurses in China’s six central and southern provinces, including Henan, Hubei, Hunan, Guangxi, Guangdong, and Hainan. A total of 55 hospitals (30 tertiary hospitals and 25 secondary and lower hospitals) were sampled through stratified sampling. There are three levels of hospitals in China based on factors such as hospital scale, number of beds, and technical level, and tertiary hospitals are the highest level among them (http://www.nhc.gov.cn/). Although the hospital scale and mission of the three types of hospitals are different, the main job duties and directions of oncology nurses in them are the same. We contacted the nursing director of each hospital and sent the study materials. After soliciting their cooperation, the nursing director of the corresponding hospital distributed the survey link to the potential participant nurses via WeChat, a popular social media almost used by all nurses in China. The inclusion criteria were (a) informed consent, (b) being a registered nurse providing direct care, (c) working in an oncology department or tumor specialty, and (d) having worked in the oncology department for more than six months [12]. The exclusion criteria were (a) not having continuous work during the investigation and (b) suffering from severe physical or mental illness. Based on the empirical power tables, a sample size of at least 558 is required to achieve a power of 0.8 when testing for mediating effects using the percentile bootstrap method [36].

### Data collection

Data were collected between March and June 2021. Points considered included that the hospitals were distributed, the nurses were on a shift rotation, and some of the questions may have been sensitive. Thus, we conducted an anonymous online survey on WeChat, a popular social media in China, to ensure that the participants were not restricted by time and place. In addition, we do not collect any personally identifiable information such as the names of participants to maintain privacy. Of the 2800 returned questionnaires, 2530 were valid for analysis, and the effective return ratio was 90.4%.

### Measures

#### Sociodemographic variables

The researchers designed the sociodemographic variables of the participants for the study, including work unit, hospital level, gender, age, working years, working years in the oncology department, first degree, highest degree, professional title, marital status, and birth condition. These variables were treated as confounding variables in this study.

#### Depression

Depression was assessed as an independent variable with the Self-Rating Depression Scale (SDS). Zung [37] developed the SDS to determine the severity of depressive mood, symptoms, or states. The scale consists of 20 items, 10 of which are positively scored and ten reverse scored. The scale uses a 4-point Likert scoring system (1 point means “no or very little time,” while 4 points represent “most or all time”). The total item score is the raw score for the SDS, and the standard score is rounded by 1.25. An SDS standard score ≥ 53 indicates depression. Higher subjects’ overall standard scores indicate higher levels of depression. The Cronbach’s α of the SDS was 0.880, which showed good reliability in Chinese hospital nurses [38]. In the present study, Cronbach’s α of the scale was 0.902.

#### Self-efficacy

Self-efficacy was the mediating variable in this study and was assessed using the General Self-Efficacy Scale (GSES) as translated and revised by Wang et al. [39] in 2001. There are ten items in the GSES, using a 4-point Likert scale. Each item is rated from 1 to 4 (1 point represents “not at all,” 2 points represent “somewhat true,” 3 points mean “primarily true,” and 4 points illustrate “absolutely true”). The scale’s total score ranges from 10 to 40; the higher the score, the higher the confidence. Previous Cronbach’s α coefficient of the GSES Chinese version was 0.870 [39].

#### Professional values

Professional values were examined as outcome variables and assessed with the Nurses Professional Values Scale (NPVS-R). The NPVS-R was compiled by Weis et al. [40] and translated and revised by Chen Tianyan [41], and consists of four dimensions: care and provide, activism, trust, and reliability, freedom, and safety. Among its 26 items, ten items regarding care reflect nurses’ perceptions of their professional competence; eight items loading onto activism emphasize nurses’ obligations to the nursing profession and society; three items regarding trust reflect the trust of patients in the medical staff; and the reliability, freedom, and safety factor reflects nurses’ protection of patients’ rights, consisting of five items. A 5-level Likert-type scoring method was adopted for each item, ranging from 1 (not important) to 5 (most important). The higher the score is, the higher the individual’s recognition of professional values. The total Cronbach’s α of the Chinese version (NPVS-R) scale was 0.760, the Cronbach’s α of each dimension was 0.663–0.796, and the retest reliability was 0.639, demonstrating good reliability and validity [41]. The scale has been widely used in the nurse population [3], and Cronbach’s α in the current study was 0.990.

### Ethical considerations

The hospitals’ ethical review boards approved this study prior to data collection. This study was anonymous; participants were informed of the purpose and methods of the study and agreed to participate by clicking the “Agree” button in the online survey. Additionally, participants realized that their participation was voluntary and confidential. They also had the right to withdraw from filling out the questionnaire at any time.

### Data analysis

SPSS 25.0 statistical packages and the PROCESS macro version 3.4.1 were employed for data analysis. Frequencies, percentages, means, and standard deviations were used for descriptive analysis, and Pearson correlation analysis was applied to explore the relationship between variables. The independent variable acts on the dependent variable through the mediating variable, and the effect produced by the mediating variable is called the mediating effect [42]. The mediating effect was identified using Hayes’s [43] Model 4 and bootstrapping analysis, and the sociodemographic control variables were determined by independent samples t-test or one-way analysis of variance for intergroup comparison. Statistical significance for all analyses was set at *P* < 0.05 (2-tailed).

## Results

### Descriptive characteristics

Table 1 shows the sociodemographic characteristics of the participants. Most participants were from tertiary hospitals (69.4%) and general hospitals (89.3%), approximately 98.5% of participants were female, and 73.0% of the nurses were aged 35 or below. For years of work, approximately 23.0% had worked for less than five years, but only 46.0% had worked in the oncology department for more than five years. For educational level, the first degree awarded was mainly junior college (52.9%), 73.3% of the participants’ highest degree was an undergraduate degree, and earning a master’s degree or higher accounted for 3.7%. Most participants had a professional title of “senior nurse” or higher level (86.1%). Moreover, 72.3% of the nurses were married, and only 33.6% were childless.

**Table 1:** Sociodemographic characteristics of nurses (*N* = 2530)

Characteristics	Category	*n* (%)	Self-efficacy		Professional values	
			*F/t*	*P*	*F/t*	*P*
Work unit						
	Specialized hospital	271 (10.7)	1.162	0.245	-1.055	0.292
	General hospital	2259 (89.3)				
Hospital level						
	Tertiary hospital	1756 (69.4)	6.714	**0.001**	21.985	**0.000**
	Secondary Hospital	691 (27.3)				
	Other	83 (3.3)				
Gender						
	Male	39 (1.5)	-0.973	0.331	1.318	0.195
	Female	2491 (98.5)				
Age (years)						
	≤ 25	423 (16.7)	3.797	**0.004**	1.134	0.339
	26–30	675 (26.7)				
	31–35	749 (29.6)				
	36–40	334 (13.2)				
	> 40	349 (13.8)				
Working years						
	≤ 5	583 (23.0)	5.769	**0.000**	0.742	0.563
	6–10	807 (31.9)				
	11–15	571 (22.6)				
	16–20	250 (9.9)				
	> 20	319 (12.6)				
Working years in the oncology department						
	< 1	558 (22.1)	7.516	**0.000**	2.433	**0.045**
	1–5	808 (31.9)				
	6–10	786 (31.1)				
	11–15	258 (10.2)				
	> 15	120 (4.7)				
First degree						
	Secondary	730 (28.9)	3.101	**0.026**	1.312	0.269
	Junior	1338 (52.9)				
	Undergraduate	459 (18.1)				
	Master’s or higher	3 (0.1)				
Highest degree						
	Secondary	42 (1.7)	9.789	**0.000**	28.569	**0.000**
	Junior	541 (21.4)				
	Undergraduate	1854 (73.3)				
	Master’s or higher	93 (3.7)				
Professional title						
	Junior nurse	352 (13.9)	8.784	**0.000**	12.788	**0.000**
	Senior nurse	995 (39.3)				
	Supervisor nurse	1001 (39.6)				
	The associate chief nurse or higher	182 (7.2)				
Marital status						
	Married	1828 (72.3)	20.025	**0.000**	36.048	**0.000**
	Singer	654 (25.8)				
	Windowed or divorced	48 (1.9)				
Birth condition						
	One child	956 (37.8)	4.793	**0.008**	3.861	**0.021**
	Two or more children	725 (28.7)				
	No children	849 (33.6)				

Percentage sums of categorical variables may not equal 100% due to rounding; Bolded means *P* < 0.05.

### Correlation analyses

The descriptive statistics and correlations among depression, self-efficacy, and professional values and their subscales are presented in Table 2. The results showed that the scores for depression, self-efficacy, and professional values of Chinese oncology nurses were 52.75 ± 12.62, 28.39 ± 6.33, and 101.55 ± 20.43, respectively. The mean scores of the items were (2.64 ± 6.31), (2.84 ± 0.63), and (3.91 ± 0.79). A total of 55.2% of Chinese oncology nurses were depressed. Furthermore, per the NPVS-R, the score of the care and provide dimension was 39.16 ± 7.94, that of the activism dimension was 30.81 ± 6.34, that of the trust dimension was 11.97 ± 2.48, and that of the reliability, freedom, and safety dimension was 19.62 ± 4.02. The mean scores of the entries for the four dimensions were (3.92 ± 0.79), (3.85 ± 0.99), (3.99 ± 0.83), and (3.93 ± 0.80). The study also found that depression was negatively related to self-efficacy (*r* = -0.252, *P* < 0.01) and professional values (*r* = -0.435, *P* < 0.01), while self-efficacy was positively correlated with professional values (*r* = 0.532, *P* < 0.01).

**Table 2:** Correlations among depression, self-efficacy, and professional values (*N* = 2530)

**Items**	**Mean (SD)**	**Items’** **Mean (SD)**	**DP**	**SE**	**PV**	**CP**	**AC**	**TS**	**RFS**
DP	52.75(12.62)	2.64(6.31)	1						
SE	28.39(6.33)	2.84(0.63)	-0.252**	1					
PV	101.55(20.43)	3.91(0.79)	-0.435**	0.532**	1				
CP	39.16(7.94)	3.92(0.79)	-0.426**	0.525**	0.992**	1			
AC	30.81(6.34)	3.85(0.99)	-0.440**	0.551**	0.983**	0.962**	1		
TS	11.97(2.48)	3.99(0.83)	-0.392**	0.456**	0.954**	0.950**	0.898**	1	
RFS	19.62(4.02)	3.93(0.80)	-0.430**	0.516**	0.983**	0.962**	0.961**	0.935**	1

Abbreviations: SD, Standard deviation; DP, Depression; SE, Self-efficacy; PV, Professional values; CP, Care and provide; AC, Activism; TS, Trust; RFS, Reliability, freedom, and safety.

**P < 0.01.

### Mediating effects of self-efficacy

In this study, bootstrapping analysis by the PROCESS macro was performed to illustrate the mediating role of self-efficacy between depression and professional values while controlling for confounding variables. The analysis of confounding variables was conducted through independent samples t-test and one-way analysis of variance, with sociodemographic variables as independent variables and self-efficacy and professional values as dependent variables. The control variables of the model were hospital level, years worked in the oncology department, highest degree, professional title, marital status, and birth condition (see Tables 1 and 3).

Table 3 presents testing results for a mediation effect through 5000 bootstrap samples. The results showed that depression had a significant predictive effect on professional values. The direct predictive effect of depression on professional values was still significant when self-efficacy was included. The results showed that depression and self-efficacy significantly predicted professional values (*β* = -0.656, *P* < 0.01 and *β* = 1.418, *P* < 0.01, respectively). Meanwhile, in the model including self-efficacy, the effect of depression on professional values was decreased (*β* = -0.493, *P* < 0.01). Furthermore, depression significantly predicted self-efficacy (*β* = -0.115, *P* < 0.01). These results indicated that self-efficacy partially mediated the relationship between depression and professional values.

**Fig. 1:**
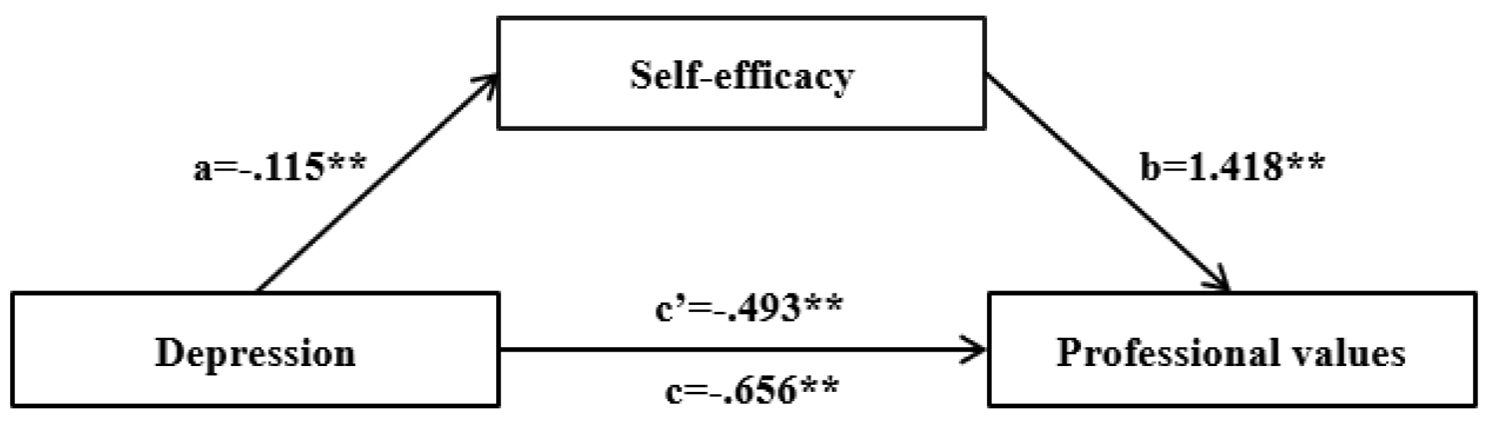
The mediating effect model of oncology nurses’ self-efficacy between depression and professional values Abbreviations: **a,** the effect of depression on self-efficacy; **b,** the effect of self-efficacy on professional values; **c,** the total effect of depression on professional values; **c’**, the direct effect of depression on professional values; **ab,** the mediating effect of self-efficacy. **P < 0.01.

In addition, the 95% bootstrapped confidence interval of the direct effect of depression on professional values and the mediating effect of self-efficacy did not contain 0 (95% Boot CI = [-0.544, -0.441]; [-0.197, -0.131], see Table 4). It appears that depression could directly predict professional values through self-efficacy’s mediating effect. The relationship between variables is shown in Fig. 1. The direct effect and the mediating effect were −0.493 and −0.163, respectively, and the mediating effect accounted for 24.8% of the total effect.

**Table 3:** The mediating model of self-efficacy between depression and professional values (*N* = 2530)

Outcome variable	Predictor variable	*R*	*R* ^**2**^	*F(df)*	*β*	*t*
Professional values						
	Depression	0.469	0.220	101.409	-0.656	-22.536**
	Hospital level				-3.220	-4.748**
	Working years in the oncology department				0.262	0.662
	Highest degree				4.693	6.435**
	Professional title				-1.011	-1.813
	Marital status				-3.957	-4.208**
	Birth condition				1.364	2.482*
Self-efficacy						
	Depression	0.282	0.080	31.199	-0.115	-11.753**
	Hospital level				-0.514	-2.253*
	Working years in the oncology department				0.414	3.108**
	Highest degree				0.629	2.563*
	Professional title				-0.050	-0.256
	Marital status				-0.987	-3.119**
	Birth condition				0.327	1.767
Professional values						
	Depression	0.630	0.398	207.872	-0.493	-18.752**
	Self-efficacy				1.418	27.276**
	Hospital level				-2.490	-4.174**
	Working years in the oncology department				-0.325	-0.933
	Highest degree				3.801	5.922**
	Professional title				-0.943	-1.924
	Marital status				-2.557	-3.089**
	Birth condition				0.900	1.863

*P < 0.05; **P < 0.01.

**Table 4:** Decomposition table of total effect, direct effect, and mediating effect (*N* = 2530)

	Effect	Boot SE	95% Boot LLCI	95% Boot ULCI	The relative effect (%)
Total effect	-0.656**	0.029	-0.713	-0.599	
Direct effect	-0.493**	0.026	-0.544	-0.441	75.2
Mediating effect	-0.163**	0.017	-0.197	-0.131	24.8

Abbreviations: Boot SE, bootstrap standard error; 95% Boot LLCI, the lowest level of the 95% Bootstrap confidence interval; 95% Boot ULCI, the highest level of the 95% Bootstrap confidence interval.

**P < 0.01.

## Discussion

In this study, we found that 55.2% of the oncology nurses were depressed, significantly higher than the 43.83% reported in the Chinese nursing population [44]. This may be related to the higher number of oncology patients and the increased pressure on oncology nurses in recent years. When patients’ conditions worsen, they show great physical and mental pain and suffering, which becomes a vital source of stress and makes nurses depressed [17, 18]. The findings suggest that managers must be concerned about depression among oncology nurses. Many studies have investigated the mental health and burnout of oncology nurses, but few studies have directly identified depression in oncology nurses as the study did. This may be a highlight of our paper.

The self-efficacy score of oncology nurses was higher than Hu et al.’s [45] survey of oncology nurses. However, the level of self-efficacy was similar to that of acute-care nurses and was significantly below the scale standard [46]. This indicates that the self-efficacy of oncology nurses is better than before but still at a lower level. The development of oncology nursing requires increasing knowledge and technical specialization of oncology nurses. Oncology specialist nurses become the development direction, with specific educational and title improvement requirements [47, 48]. In this study, undergraduate (or higher) and senior nurses (or higher) accounted for 77.0% and 86.1% of the sample, respectively, which implies that their level of expertise and nursing skills may be higher, the better their sense of value experience, and thus the self-confidence of oncology nurses has increased compared to before [49]. However, multiple factors, such as work pressure and difficulties endured by oncology nurses, have affected their confidence to some extent and reduced their self-efficacy. It is suggested that nursing managers pay attention to oncology nurses’ self-efficacy levels, actively guide and improve their self-efficacy, and enhance their work confidence [50].

Nurses’ professional values are the basis of nursing practice, underpinned by nursing staff providing safe, humane, and ethical health care and health promotion behavior for patients [6]. Satisfaction with subjective perceptions of professional values affects their work engagement and satisfaction [9]. As a result, nurses’ intention to stay and their propensity to leave can be predicted [51]. The professional values score of oncology nurses in this study was 101.55 ± 20.43, with a mean entry score of 3.91 ± 0.79, which indicates that Chinese oncology nurses’ professional values are generally intermediate. The results were lower than those of the Iranian study on the professional values of clinical nurses [52]. This may be related to the fact that nurses’ professional values can differ across social backgrounds and organizational cultures [53–55]. Firstly, the nursing activities of Chinese oncology nurses are still limited by doctors’ orders, and the phenomenon of “medical care over nursing” is still common in society, which can put pressure on them and affect their professional values [56]. Secondly, this Iranian study was conducted in a medical university hospital, which may have a stronger professional atmosphere.

Additionally, among the dimensions, the level of different dimensions of nurses’ professional values was uneven. The mean score of trust was the highest, which indicated that the oncology nurses earned respect and understanding of patients through their medical knowledge and professional skills. However, there were differences with the study findings by Guan et al. [57], which found that the dimension “reliability, freedom, and safety” was the highest. This may be explained by the fact that the tension in the nurse-patient relationship influenced the expectation of oncology nurses regarding the trust of patients and healthcare professionals. The lowest score was found for activism, similar to the findings of Wang et al. [58]. It is implied that oncology nurses have relatively little planning to enhance their professional competence level and promote their career development [13]. This may be related to heavy clinical workloads that make nurses less likely to participate in other activities. Nursing managers should make reasonable allocations of nursing human resources according to the current situation and the influencing factors of nurses’ professional values. There is a need to strengthen nurses’ support systems, provide targeted training and guidance, and help nurses establish positive nursing professional values.

This study showed that depression was negatively associated with self-efficacy, which is consistent with previous studies [27, 59]. This means that the higher the level of depression, the lower the level of self-efficacy. The literature showed that the higher the depressive symptom score, the higher the negative attributions; consequently, the more prominent the self-denial, the lower the degree of self-acceptance and self-evaluation, followed by a decrease in self-efficacy [60]. Therefore, the self-efficacy of oncology nurses could be improved by alleviating their depression. In addition, correlation analysis found that the total professional values score of oncology nurses was negatively associated with depression and positively correlated with self-efficacy. Previous research has pointed out that oncology nurses with high professional values had low levels of depression and high levels of self-efficacy [61]. Managers can improve the professional values of nursing staff by enhancing their psychological situation or increasing their self-efficacy to guide their positive behavioral performance [14, 28].

Among the dimensional correlations, the dimensions of professional values were positively correlated with each other. The higher the professional competence of the nurse, the higher the professional trust of the patient, and the more practical action the nurse can afford to provide care and protection for the patient’s rights. Moreover, depression was negatively related to all dimensions of professional values. Oncology nurses in a depressed state provided poorer patient care and practice activities, as well as lower trust, reliability, freedom, and safety [21, 62]. In contrast, self-efficacy was positively correlated with all dimensions of professional values. It is suggested that nursing managers improve nurses’ self-efficacy to develop their proper professional values [30].

Mediating effects analysis explains the processes and mechanisms of influence between variables and can generate more in-depth results than regression analysis [63]. The results of the correlation analysis showed that oncology nurses’ self-efficacy, depression, and professional values were correlated, so this study met the conditions of mediating effect analysis.

The mediating effect analysis showed that the mediation effect of oncology nurses’ self-efficacy between depression and professional values was significant, with the proportion of the mediation effect being 24.8%. On the one hand, the findings indicate that depression has a direct negative impact on the professional values of nurses. This indicates that the higher the degree of depression of nurses, the lower their professional values. On the other hand, our research found that nurses’ depression indirectly affects their professional values through their self-efficacy. Furthermore, to our knowledge, this interesting discovery has not been previously made. The higher the level of depression, the lower the nurses’ self-efficacy, which also harms the professional values of nurses. This effect level corresponds to 24.8% of the influence of depression on nurses’ professional values. Analysis of the reasons for this suggests the following: self-efficacy is a subjective judgment to reflect whether a nurse is competent for nursing work [25]. The higher the nurse’s self-efficacy level, the stronger the self-confidence to complete clinical work. The more comfortable they are in their work; the less likely depression will occur [27]. Additionally, they tend to be more enthusiastic about their work and have a higher sense of professional values because of their relatively relaxed mood [29].

These results suggest that clinical nursing managers can enhance oncology nurses’ professional values and self-efficacy by focusing on their psychological status and alleviating their depression. They could also pay attention to the cultivation of nurses’ self-efficacy and implement targeted measures to carry out relevant education and effectively improve the professional values of nurses to benefit the development of clinical nursing.

### Strengths and Limitations

Our study first explored the relationship between depression, self-efficacy, and professional values among Chinese oncology nurses with a sufficient sample size. Although novel, there were still some limitations. First, this study was a cross-sectional survey that cannot explain causality. Further longitudinal studies are needed to explore the relationships among these variables across time. Second, we used self-report tools. Further studies may consider adding physiological markers such as salivary cortisol to measure depression to enhance the objectivity of the assessment.

## Conclusions

In summary, we have revealed that depression can negatively predict self-efficacy and professional values and that self-efficacy can positively predict professional values. Meanwhile, self-efficacy can partially mediate the relationship between depression and professional values among Chinese oncology nurses. The findings suggest that the effect of depression on professional values can be reduced by improving self-efficacy, which provides a new reference for enhancing the development of professional values strategies for oncology nurses.

## Electronic supplementary material

Below is the link to the electronic supplementary material.

Additional file 1. STROBE Statement

## Data Availability

The data that support the findings of this study are available on request from the corresponding author.
